# Sphingosine-1-phosphate receptor-2 facilitates pulmonary fibrosis through potentiating IL-13 pathway in macrophages

**DOI:** 10.1371/journal.pone.0197604

**Published:** 2018-05-21

**Authors:** Juanjuan Zhao, Yasuo Okamoto, Yuya Asano, Kazuhiro Ishimaru, Sho Aki, Kazuaki Yoshioka, Noriko Takuwa, Takashi Wada, Yutaka Inagaki, Chiaki Takahashi, Takumi Nishiuchi, Yoh Takuwa

**Affiliations:** 1 Department of Physiology, Kanazawa University School of Medicine, Ishikawa, Japan; 2 Department of Health and Medical Sciences, Ishikawa Prefectural Nursing University, Ishikawa, Japan; 3 Department of Nephrology and Laboratory Medicine, Kanazawa University School of Medicine, Ishikawa, Japan; 4 Center for Matrix Biology and Medicine, Graduate School of Medicine, Tokai University, Kanagawa, Japan; 5 Division of Oncology and Molecular Biology, Cancer Research Institute, Kanazawa University, Ishikawa, Japan; 6 Division of Functional Genomics, Advanced Science Research Center, Kanazawa University, Ishikawa, Japan; University of Pittsburgh, UNITED STATES

## Abstract

Idiopathic pulmonary fibrosis is a devastating disease with poor prognosis. The pathogenic role of the lysophospholipid mediator sphingosine-1-phosphate and its receptor S1PR2 in lung fibrosis is unknown. We show here that genetic deletion of *S1pr2* strikingly attenuated lung fibrosis induced by repeated injections of bleomycin in mice. We observed by using *S1pr*2^LacZ/+^ mice that S1PR2 was expressed in alveolar macrophages, vascular endothelial cells and alveolar epithelial cells in the lung and that S1PR2-expressing cells accumulated in the fibrotic legions. Bone marrow chimera experiments suggested that S1PR2 in bone marrow–derived cells contributes to the development of lung fibrosis. Depletion of macrophages greatly attenuated lung fibrosis. Bleomycin administration stimulated the mRNA expression of the profibrotic cytokines IL-13 and IL-4 and the M2 markers including arginase 1, Fizz1/*Retnla*, Ccl17 and Ccl24 in cells collected from broncho-alveolar lavage fluids (BALF), and *S1pr2* deletion markedly diminished the stimulated expression of these genes. BALF cells from bleomycin–administered wild-type mice showed a marked increase in phosphorylation of STAT6, a transcription factor which is activated downstream of IL-13, compared with saline–administered wild-type mice. Interestingly, in bleomycin–administered *S1pr2*^-/-^ mice, STAT6 phosphorylation in BALF cells was substantially diminished compared with wild-type mice. Finally, pharmacological S1PR2 blockade in *S1pr2*^*+/+*^ mice alleviated bleomycin–induced lung fibrosis. Thus, S1PR2 facilitates lung fibrosis through the mechanisms involving augmentation of IL-13 expression and its signaling in BALF cells, and represents a novel target for treating lung fibrosis.

## Introduction

Idiopathic pulmonary fibrosis (IPF) is a progressive and ultimately fatal disease with a poor median survival of 2–3 years after diagnosis [1–3]. IPF is characterized by deposition of extracellular matrix proteins including collagens and fibronectin and fibroblast proliferation, which result in chronic pathological remodeling of lung and eventually respiratory failure. IPF is likely elicited by persistent injury of alveolar epithelial and vascular endothelial cells, which is accompanied by the infiltration of leukocytes into injured sites. These damaged cells and infiltrating leukocytes release inflammatory cytokines and profibrogenic factors such as interleukin (IL)-33, IL-4, IL-13 and transforming growth factor-β (TGFβ), which may act in linear or parallel pathways. The cytokines and profibrogenic factors activate resident fibroblasts to induce differentiation into myofibroblasts, which robustly produce extracellular matrix proteins to form fibrotic lesions [4]. Particularly, the importance of IL-4 and IL-13, which are T helper type 2 cytokines and act mainly through phosphorylating STAT6, are increasingly recognized as the critical profibrogenic mediators in lung fibrosis [1–4]. In addition, bone marrow (BM)–derived circulating fibrocytes, and pericytes–and epithelial cells–derived fibroblasts may also contribute to myofibroblasts in fibrotic lesions. Although the pathogenesis of IPF has been extensively studied, the precise molecular and cellular pathogenesis for IPF is not fully understood and effective therapeutic strategies are still lacking.

Sphingosine-1-phosphate (S1P) is a pleiotropic lysophospholipid mediator and regulates various biological processes such as embryonic development, vascular formation, vascular barrier integrity and lymphocyte trafficking via five members of S1P–specific G protein–coupled receptors (S1PR1–S1PR5) [5]. Several lines of evidence suggested the possible involvement of S1P signaling in lung fibrosis [6, 7]. S1P increased the expression of the myofibroblast marker α-smooth muscle actin and collagen synthesis in cultured fibroblasts [8, 9]. Knockdown of either S1PR2, S1PR3 or the S1P–synthesizing enzyme sphingosine kinase-1 (SphK1) inhibited TGFβ–induced myofibroblast differentiation in vitro, suggesting the participation of S1P signaling in the profibrotic actions of TGFβ [10]. In mouse models of lung fibrosis, S1PR1 was shown to inhibit lung fibrosis [11] whereas S1PR3 mediated the development of lung fibrosis [12]. In patients with IPF, S1P concentrations in serum and bronchoalveolar lavage fluid (BALF) were elevated with the increased expression of SphK1 in the lung tissues [13].

Among S1PRs, S1PR2 exerts a unique inhibitory effect on migration of monocytes/macrophages [14] and mast cells [15], a facilitating effect of myofibroblast differentiation [8, 9, 16], and a protective effect on vascular barrier integrity [17]. Hence, we focused on S1PR2 in this study that aimed at revealing a role of S1P signaling in lung fibrosis. We found that genetic deletion of *S1pr2* dramatically inhibited bleomycin–induced lung fibrosis through the mechanisms involving the potentiation of the responses to the profibrotic cytokine IL-13. These findings suggest that targeting S1PR2 may offer a novel therapeutic strategy for lung fibrosis.

## Materials and methods

### Reagents

Rabbit monoclonal anti–Tyr^641^–phosphorylated STAT6 (p-STAT6) antibody (ab54461) and rabbit monoclonal anti–STAT6 antibody (ab32520) were bought from Abcam (Cambridge, MA). Rabbit monoclonal anti–Tyr^701^–phosphorylated STAT1 (p-STAT1) antibody (#9167) and rabbit polyclonal anti–STAT1 antibody (#9172) were bought from Cell Signaling Technology (Beverly, MA). Goat polyclonal anti–platelet–derived growth factor (PDGF) receptor α antibody (AF1062) was bought from Roche (Nutley, NJ). Rat monoclonal anti-Mac3 antibody (550292) was bought from BD Pharmingen (San Diego, CA). Bleomycin was bought from Nippon Kayaku (Tokyo, Japan). 4-[2((1R,2R)-2-Hydroxycyclohexylamino)-benzothiazol-6-yloxyl]-pyridine- 2-carboxylic acid methylamide (BLZ945) was purchased from Chemshuttle (Jiangsu, China). Rat monoclonal IgG_2a_ anti-mouse IL-13 antibody (38213) and control rat monoclonal IgG_2a_ (54447) were bought from R&D systems (Minneapolis, MN). 4’6-diamidino-2-phenylindole (DAPI) (D1306) was purchased from Molecular Probes (Eugene, OR). FastStart Universal SYBR Green Master was bought from Roche (Nutley, NJ). All other reagents were bought from Wako (Osaka, Japan) unless otherwise specified. An S1PR2–specific antagonist (S1PR2i) [18] was provided by Ono Pharmaceutical Co.

### Mice

*S1pr2*–null mice with targeted disruption of the *S1pr2* gene (*S1pr2*^*-/-*^) (129Ola;C57BL/6J mixed background) that had been backcrossed to C57BL/6J mice (Charles River) once were previously described [19]. *S1pr2*^-/-^ and control wild-type littermate mice were obtained using a heterozygous breeding strategy, and the latter mice were employed as appropriate controls. *S1pr2*^LacZ/+^ mice in which LacZ is knocked in at the *S1pr2* locus (129Ola;C57BL/6J mixed background) were more than 10 times backcrossed to C57BL/6J mice [19]. *Col1α2–EGFP* transgenic mice [20] were mated to *S1pr2*^*-/-*^ mice to generate *S1pr2*^*-/-*^; *Col1α2–*E*GFP* mice. Mice were housed in a temperature-controlled conventional facility (24°C) under a 12:12 h light–dark cycle with free access to regular chow and water. Mice were sacrificed at each observational time point using intraperitoneal pentobarbital (Kyoritsu, Tokyo, Japan) overdose according to the acceptable *euthanasia* guidelines. Mice were genotyped by PCR analysis of genomic DNA prepared from tail biopsies. All animal experiments were conducted according to the Fundamental Guidelines for Proper Conduct of Animal Experiment and Related Activities in Academic Research Institutions under the jurisdiction of the Ministry of Education, Culture, Sports, Science and Technology of Japan, and were approved by the Committee on Animal in Kanazawa University. We did not perform a priori power analyses, and the sample sizes were chosen in this study so that the sample sizes became similar to those generally employed in the field. In addition, all efforts were made to minimize animal suffering and to reduce the number of animals required.

### Bleomycin-induced lung fibrosis model

To induce lung fibrosis, mice (8–10 week-old, 20–25 g body weight) were challenged with i.p. injections of bleomycin at 0.035 U/g twice weekly for 4 weeks [21, 22]. Control animals received intraperitoneal 150 μl sterile 0.9% saline. Mice were euthanized for analysis 1 week after the final injection. During the period of bleomycin administration *S1pr2*^*-/-*^ and wild-type mice showed 15% and 23% reductions in the average values of body weight, respectively. In contrast, the saline groups of *S1pr2*^*-/-*^ and wild-type mice showed 5% and 9% increases in body weight. For administration of BLZ945, BLZ945 was dissolved in 20% Captisol at a concentration of 12.5 mg/ml and given to mice at the dose of 200 mg per kg body weight by oral gavage once daily [23]. In parallel, control mice received 20% Captisol or none. For neutralization of IL-13, mice received 5 mg per kg body weight of monoclonal anti-IL-13 antibody or control IgG in Dulbecco’s phosphate buffered saline (PBS) by i.p. injection every 11 days, beginning 1 day before starting bleomycin injection. For administration of S1PR2i to mice, S1PR2i was powdered and mixed into the powder diet at 0.06% (wt/wt) [18]. Mice were fed the powder diet with or without S1PR2i *ad libitum*, beginning 3 days before starting bleomycin administration. The amounts of S1PR2i administered into mice were calculated from weights of ingested powder diet.

### Determination of fibrotic regions

Mice were euthanized by pentobarbital overdose and the lungs were quickly inflated by injecting 0.5% low-melting point agarose (Agarose XP) (Wako) and 4% paraformaldehyde (PFA) in PBS into the trachea via a blunted 18-gauge needle. Isolated lungs were embedded in paraffin. Sections were cut 5-μm thick, followed by staining with Sirius Red to assess extents of fibrotic deposition, as described [24]. To quantitate Sirius Red staining–positive pixels, images of three sagittal sections through hilar levels, central levels and peripheral levels per each left whole lung were acquired at 4x objective magnification using BZ-II Analyzer software (Keyence, Osaka, Japan) on a BIOREVO BZ-9000 microscope (Keyence, Osaka, Japan). Images were then converted into 8-bit grey-scale TIFF files using the ImageJ software (NIH ImageJ; http://rsbweb.nih.gov/ij/). Areas of parenchyma were manually outlined to exclude airways and large vessels and then thresholded with visual comparison being made to the images to ensure that the tool effectively resolved Sirius Red–stained fibrotic lesions. Data were expressed as fibrotic region versus total lung area without airways and large vessels. Fibrotic regions were determined in all of the three sagittal sections per each left lung, and finally the mean value of the determinations of the three sections was calculated and taken as the fibrotic score of each mouse. To avoid bias, evaluations were made in a blinded fashion.

### Immunohistochemistry and 5-bromo-4-chloro-3-indolyi-β-D-galactopyranoside (X-gal) staining

The deparaffinized lung sections were incubated in Target Retrieval Solution (DAKO, Carpinteria, CA) at boiling temperature for 30 min to retrieve antigen before quenching endogenous peroxidase activity with 3% H_2_O_2_ in PBS for 15 min at room temperature. The sections were blocked in Protein block Serum Free (DAKO) for 1 hour, and then incubated overnight at 4°C in goat polyclonal anti–PDGFRα antibody (1:200). Sections were then incubated in biotinylated anti-goat IgG (1:200) and in ABC reagents (Vectastain ABC kit) (Vector Laboratories, Burlingame, CA) for 1 hour at room temperature. Signals were visualized with DAB solution (0.05% DAB and 0.03% H_2_O_2_ in PBS). Parallel sections processed identically but without the incubation in the primary antibody served as negative controls. Finally, sections were washed, counterstained with hematoxylin. Image acquisition was carried out with a DP20 camera attached to a standard bright-field microscope (BX 41 (Olympus, Tokyo, Japan)). X-gal staining was prepared as previously described [19].

### Analyses of bronchoalveolar lavage fluids

Mice were anesthetized by i.p. injections of sodium pentobarbital (70 mg/kg), their tracheas were cannulated, and BALF was collected by instilling 1 ml of sterile PBS into the trachea twice via a blunted 18-gauge needle. Recovery of the fluid was consistently 70–80% of the total. For determination of total number of cells in BALF, the BALF was then centrifuged at 1,000 rpm for 10 min at 4°C, and the cell-free supernatant was collected and stored at -80°C until analysis. The cell pellets were resuspended in 1 ml PBS and counted with an automated cell counter (TC50 Automated Cell Counter) (Bio-Rad, Hercules, CA). The remaining cells were centrifuged and resuspended in a small amount of a buffer at ~10^6^ cells/μL. Five μL of the cell suspension was spotted on slide glasses, and extended with a cover glass to make a thin film of the cell suspension. Slide glasses were air-dried and stained with Diff-Quick stain (Sysmex, Kobe, Japan). The percentages of macrophages, lymphocytes and neutrophils were determined by counting a minimum of 200 cells per slide under a BX 41 bright-field microscope. Soluble collagen content in BALF was determined using the Sircol Soluble Collagen Assay kit (Biocolor, Northern Ireland, UK) according to the manufacturer’s instructions. Total protein concentrations in BALF were determined by Lowry’s method as described previously [25].

### RNA isolation and quantitative polymerase chain reaction (qPCR)

RNA isolation from BALF cells and qPCR were performed as previously described [26]. The housekeeping gene β-actin was used as an internal control. Primers are listed in S1 Table. An average threshold cycle (Ct) value was calculated from duplicate results for each RNA sample and was normalized to that of β-actin to obtain the ΔCt value.

### Bone marrow transplantation

BM chimeras were prepared as previously described with minor modifications [27]. Briefly, recipient WT and *S1pr2*^*-/-*^ mice aged 8–10 weeks were irradiated 1 day before BMT by a sublethal dosage of 4.8 Gy twice at 3-hour intervals. Bone marrow cells were collected by flushing the marrow cavity of femurs and tibias of donor WT and *S1pr2*^*-/-*^ mice. Unfractionated bone marrow cells (1 x 10^7^ cells per recipient) were injected into recipient mice via a tail vein. Analysis with a fluorescence activated cell sorter showed that peripheral blood cells of the recipients were almost completely (~90%) reconstituted with donor BM-derived cells after one month. All mice were injected with bleomycin 6 weeks after transplantation.

### Microarray

Two independent RNA samples of each genotype were used to verify reproducibility of the microarray analyses. RNA sample quality was assessed using the Tapestaion 2200 (Agilent Technologies, Santa Clara, CA). One-color microarray analysis was performed according to the Agilent 60-mer Oligo Microarray Processing Protocol (Agilent Technologies). Total RNA samples (200 ng) were used to prepare Cy3-labeled cRNA using the Low Input Quick Amp Labeling Kit (Agilent Technologies). Hybridization solutions containing fragmented cRNAs (1650 ng) were then applied to the Agilent 026655 whole mouse genome Microarray 4x44k v2 that contains 44,000 probes for mouse genes (Agilent Technologies). The hybridized and washed arrays were scanned in the Cy3 channel using an Agilent microarray scanner (G2565BA; Agilent Technologies). The images were analyzed using Feature Extraction Software (Ver. 10.7.3.1; Agilent Technologies). These data were analyzed further with GeneSpring GX12.5 software (Agilent Technologies). Normalization was performed as follows: 1, intensity-dependent Lowess normalization; 2, data transformation, with measurements less than 0.01 set to 0.01; 3, per-chip normalization, in which the 75th percentile method was used to normalize each array; 4, per-gene normalization, in which the data were normalized to control (wild-type) samples. After normalization, statistically significant gene sets were defined as those exhibiting P values below the cut-off of 0.05. Genes with a >1.5-fold change (FC) in expression ratio (*S1pr2*^*-/-*^ vs wild-type) were identified. Thus, a combination of statistical analyses and FC methods was used. The microarray data from this publication were deposited in GEO (accession number: GSE85411).

### Western blot analysis

BALF cells were treated as indicated and lysed in the radioimmunoprecipitation assay (RIPA) buffer containing 50 mM Tris-HCl (pH 7.4), 150 mM NaCl, 0.1% SDS, 0.5% deoxycholate, 1% NP-40 and cOmplete Mini (Roche, Nutley, NJ). The cell lysates were centrifuged at 15,000 rpm for 15min at 4°C and the resultant supernatants were separated on SDS-8% polyacrylamide gels, followed by electrotransfer onto Immobilon-P membrane (Millipore, Bedford, MA). The membranes were blocked with Tris-buffered saline (pH 7.4) containing 0.1% Tween-80 and 5% non-fat dry milk for 1 hour at room temperature and probed with anti–p-STAT6 antibody (1:500 dilution), anti–p-STAT1 antibody (1:500), anti–STAT6 antibody (1:500), or anti–STAT1 antibody (1:500). The bound antibodies were visualized using anti–rabbit IgG alkaline phosphatase–conjugated secondary antibody (1:1000) and 5-bromo-4-chloro-3-indolyl phosphate and nitro blue tetrazolium. Relative quantification of proteins was determined by scanning and band densitometry using Image Gauge software (Fuji Photo Film, Tokyo, Japan).

### Determinations of cytokine concentrations

To prepare lung tissue homogenates for ELISA analysis, frozen lung tissues were homogenized at 50 mg wet tissues/ml in Hank’s balanced salt solution. After being centrifuged at 800 g for 10 min, the supernatant from the lung homogenate was collected for ELISA analysis [28]. The levels of IL-13 in BALF samples were determined with mouse IL-13 Mini ELISA Development Kit (Peprotech), according to the manufacturer’s protocols.

### Statistical analysis

All experiments were repeated at least three times. All data are presented as means ± SEM. Numbers of mice and samples analyzed were indicated in the Figures. Statistical analyses were performed with Prism 6 software (GraphPad Software, La Jolla, CA) using two-way ANOVA (multiple factor analysis) or one-way ANOVA, which was followed by the Bonferroni post-test, or two-tailed Student’s t-test when comparing two groups. P < 0.05 was considered to indicate statistical significance.

## Results

### Genetic deletion of *S1pr2* prevents bleomycin-induced pulmonary fibrosis

**Fig 1:**
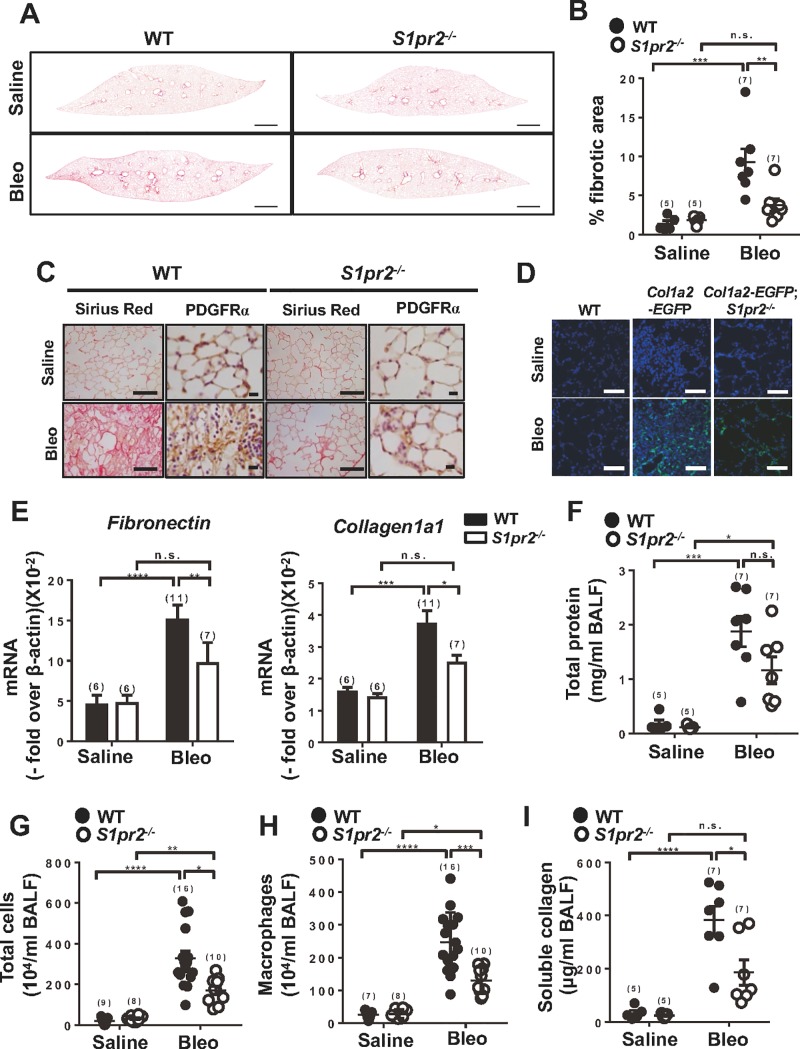
Genetic deletion of *S1pr2* prevents bleomycin–induced lung fibrosis. The lungs (A-E) and BALF (F-I) were collected from mice on day 33 after starting saline or bleomycin administration and analyzed. (A) Representative images at low magnification (x40) of Sirius Red–stained whole lung sections from saline–or bleomycin (Bleo)–administered wild-type (WT) and *S1pr2*^*-/-*^ mice. Scale bar: 600 μm. (B) Quantification of fibrotic areas in whole lung sections from WT and *S1pr2*^*-/-*^ mice. (C) Representative images at high magnification (x400) of immunostaining using anti-PDGFRα antibody and Sirius Red–stained lung sections from WT and *S1pr2*^*-/-*^ mice. Scale bar:100 μm. (D) Representative fluorescent images of EGFP fluorescence (green) and nuclear staining (DAPI, blue) in the lung from *Col1α2*−EGFP and *Col1α2*−EGFP:*S1pr2*^*-/-*^ mice. Magnification: x400. Scale bar: 50 μm. (E) mRNA expression levels of *Fibronectin* and *Collagen1a1* in the lung from WT and *S1pr2*^*-/-*^ mice. (F-I) Total protein concentrations (F), total cell numbers (G), Macrophage numbers (H) and soluble collagen levels (I) in BALF from WT and *S1pr2*^*-/-*^ mice. The individual values and the mean ± SEM are shown. N = 5−16 from three to seven independent experiments. *P <0.05, **P <0.01, ***P<0.001, ****P<0.0001 and n.s. no significance (two-way ANOVA followed by the Bonferroni post-test).

*S1pr2*^*+/+*^ and *S1pr2*^*-/-*^ littermates were subjected to repeated i.p. injections of bleomycin, which mimics chemotherapeutic regimen in human patients and induces sustained lung fibrosis [21, 22]. Bleomycin–administered *S1pr2*^*-/-*^ mice displayed attenuation of lung fibrosis compared with wild-type mice, as evaluated with collagen staining (Fig 1A and 1B). In *S1pr2*^*-/-*^ mice given bleomycin, platelet-derived growth factor receptor α (PDGFRα)–positive fibroblasts of the lung were dramatically decreased compared with wild-type mice (Fig 1C). In *Col1α2–EGFP* transgenic mice in which enhanced green fluorescent protein (EGFP) gene expression is driven by the *Col1α2* enhancer/promoter [20], bleomycin administration increased EGFP–positive cells in the lung sections (Fig 1D). Bleomycin–induced increase in EGFP–positive cells was markedly reduced in *Col1α2–EGFP;S1pr2*^*-/-*^ compound mutant mice compared with *Col1α2–EGFP* mice (*S1pr2*^*+/+*^), further supporting that *S1pr2* deletion reduced fibroblast accumulation in the lung. In addition, bleomycin–induced increases in fibronectin and collagen1α1 mRNAs in the lung were attenuated in *S1pr2*^*-/-*^ mice compared with wild-type mice (Fig 1E). Bleomycin induces inflammation in lung [29]. Bleomycin administration increased protein concentrations and inflammatory cells in BALF of wild-type mice (Fig 1F and 1G). Approximately 70–80% of the cells in BALF of bleomycin–administered mice were macrophages. Bleomycin administration increased macrophages in BALF of wild-type mice. In *S1pr2*^*-/-*^ mice given bleomycin, total cells and macrophages were reduced compared with wild-type mice (Fig 1F–1H). Bleomycin–induced increase in the soluble collagen concentration of BALF was also attenuated in *S1pr2*^*-/-*^ mice compared with wild-type mice (Fig 1I). Thus, the loss of *S1pr2* substantially suppressed pulmonary inflammation and fibrogenesis elicited by repeated administration of bleomycin.

### Expression of S1PR2 in the lung of *S1pr2*^LacZ/+^ mice

**Fig 2:**
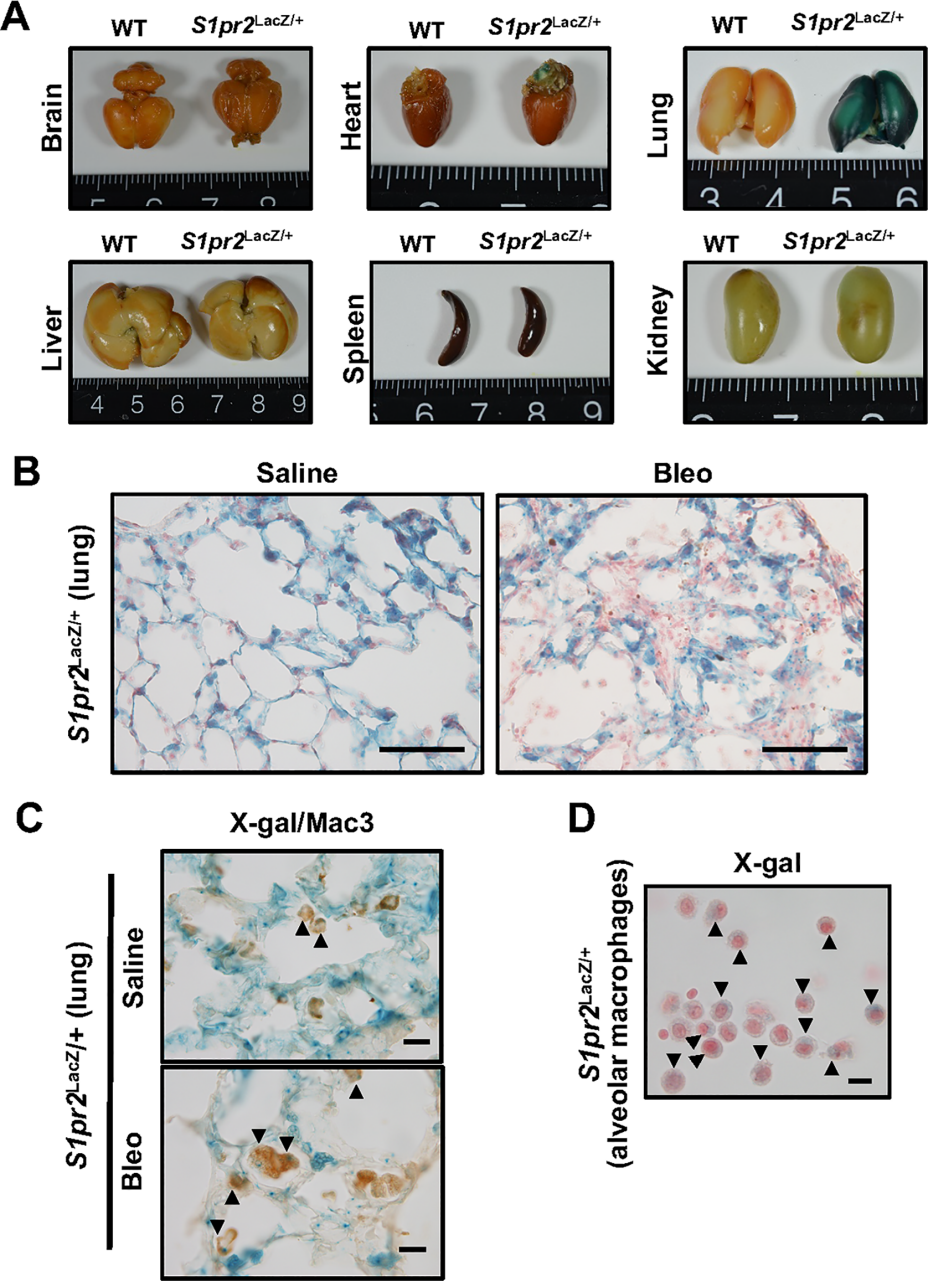
Expression of S1PR2 in the lung of *S1pr2*^LacZ/+^ mice. (A) X-gal stained brain, heart, lung, liver, spleen and kidney from 8-week old WT and *S1pr2*^LacZ/+^ mice. (B) X-gal stained lung sections of *S1pr2*^LacZ/+^ mice on day 33 after starting administration of saline or bleomycin (Bleo). Magnification: x400. Scale bar: 100 μm. (C) X-gal stained and anti–Mac3 immunostained lung sections of *S1pr2*^LacZ/+^ mice on day 33. Magnification: x1000. Scale bar: 100 μm. (D) X-gal stained alveolar macrophages in the BALF smear from bleomycin–administered *S1pr2*^LacZ/+^ mice. Magnification, x1000. Scale bar, 100 μm. In (B) and (D), the tissue sections were counterstained by nuclear fast red. In (C) and (D), the arrowheads denote X-gal-stained macrophages. The presented data represent three independent experiments.

In *S1pr2*^LacZ/+^ mice in which LacZ (*Escherichia coli* β-galactosidase) gene expression is driven by the endogenous *S1pr2* promoter [19], the lung among various organs examined was most intensely stained with X-gal chemical staining (Fig 2A), indicating that the lung abundantly expresses S1PR2. In the alveolar area of saline–administered *S1pr2*^LacZ/+^ mice, it appears that the alveolar cells and the interstitial cells were X-gal–positive (Fig 2B). In bleomycin–administered mice, X-gal–positive cells were abundant in the fibrotic lesions of lung. The double staining of anti-Mac3 immunohistochemistry and X-gal chemical staining indicated that alveolar macrophages are X-gal–positive (Fig 2C). This was also confirmed with X-gal staining of BALF smears (Fig 2D).

### *S1pr2* deletion in bone marrow–derived cells suppresses bleomycin–induced fibrosis

**Fig 3:**
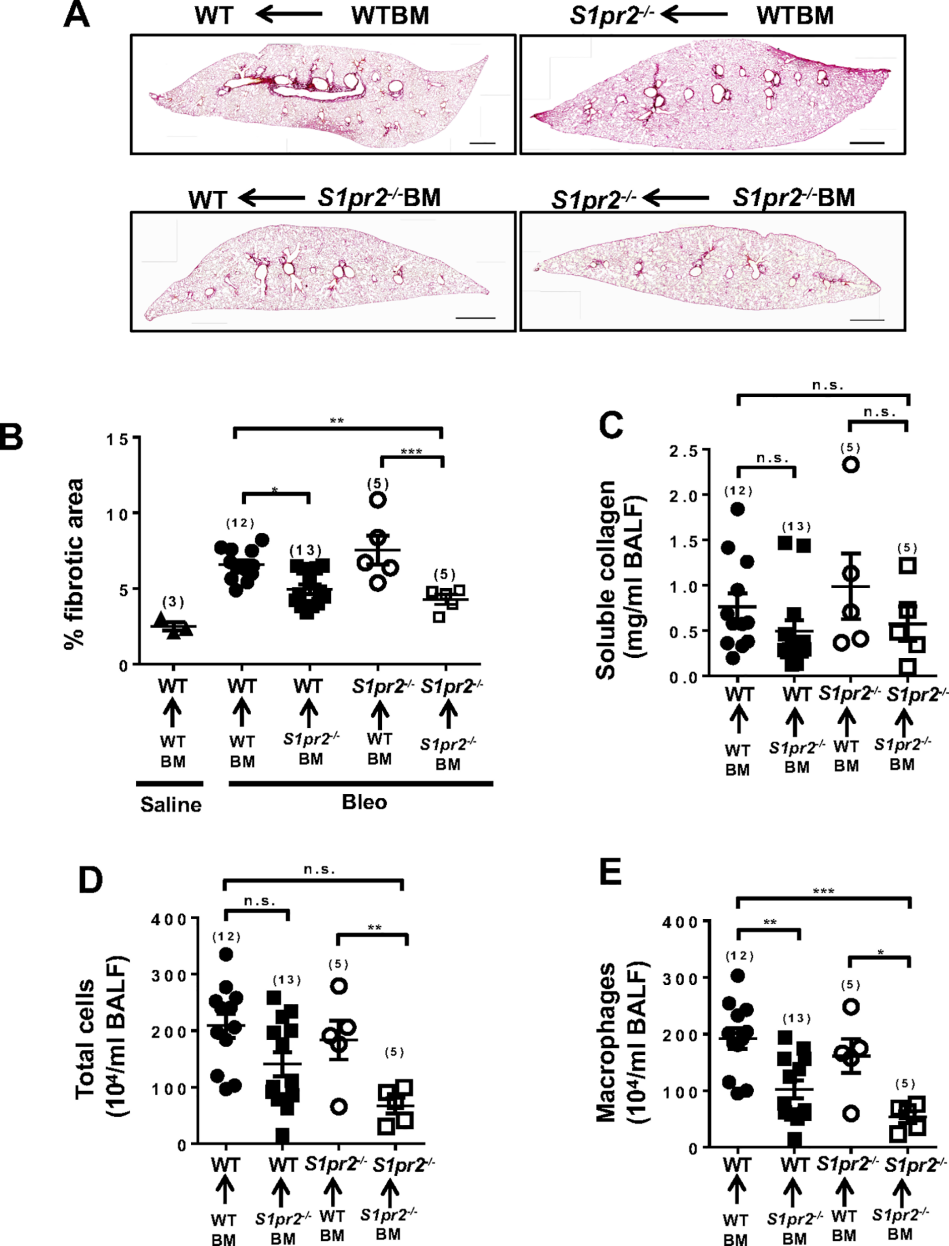
*S1pr2* deletion in bone marrow–derived cells suppresses bleomycin–induced lung fibrosis in mice. Wild-type (WT) and *S1pr2*^*-/-*^ mice had been transplanted with BM from either WT or *S1pr2*^*-/-*^ mouse donors. The lungs (A, B) and BALF (C-E) were collected from BM–chimeric wild-type or *S1pr2*^*-/-*^ mice. on day 33 after starting administration of saline or bleomycin (Bleo) and analyzed. (A) Representative images of Sirius Red–stained whole lung sections Magnification: x40. Scale bar: 600 μm. (B) Quantification of lung fibrotic lesions using image analysis of Sirius Red–stained lung sections. (C-E) Soluble collagen levels (C), total cell numbers (D), and macrophage numbers (E) in BALF. The details of BM transplantation are indicated on each image in (A) and at the bottoms of (B) to (E). The individual values and the mean ± SEM are shown. N = 5−13 from five independent experiments. *P <0.05, **P <0.01, ***P<0.001 and n.s. no significance (two-way ANOVA followed by the Bonferroni post-test).

We studied the involvement of S1PR2 in bone marrow–derived cells in bleomycin–induced fibrosis using BM chimera mice (Fig 3). Bleomycin–administered wild-type mice that received BM from *S1pr2*^*-/-*^ donor mice exhibited a reduction in the lung fibrotic area compared with wild-type mice receiving wild-type BM, while *S1pr2*^*-/-*^ mice that received BM from wild-type mice showed exaggeration of fibrosis compared with *S1pr2*^*-/-*^ mice receiving BM from *S1pr2*^*-/-*^ mice (Fig 3A and 3B). Wild-type and *S1pr2*^*-/-*^ mice that were both given wild-type BM tended to have higher soluble collagen concentrations in BALF compared with the respective counterparts receiving *S1pr2*^*-/-*^ BM, although not statistically significant (Fig 3C). Moreover, macrophages and total cells in BALF were reduced in wild-type mice receiving *S1pr2*^*-/-*^ BM compared with wild-type mice receiving wild-type BM while these parameters were elevated in *S1pr2*^*-/-*^ mice receiving wild-type BM compared with *S1pr2*^*-/-*^ mice receiving *S1pr2*^*-/-*^ BM (Fig 3D and 3E). These data are compatible with the notion that S1PR2 in BM–derived cells are involved in bleomycin–induced fibrosis.

**Fig 4:**
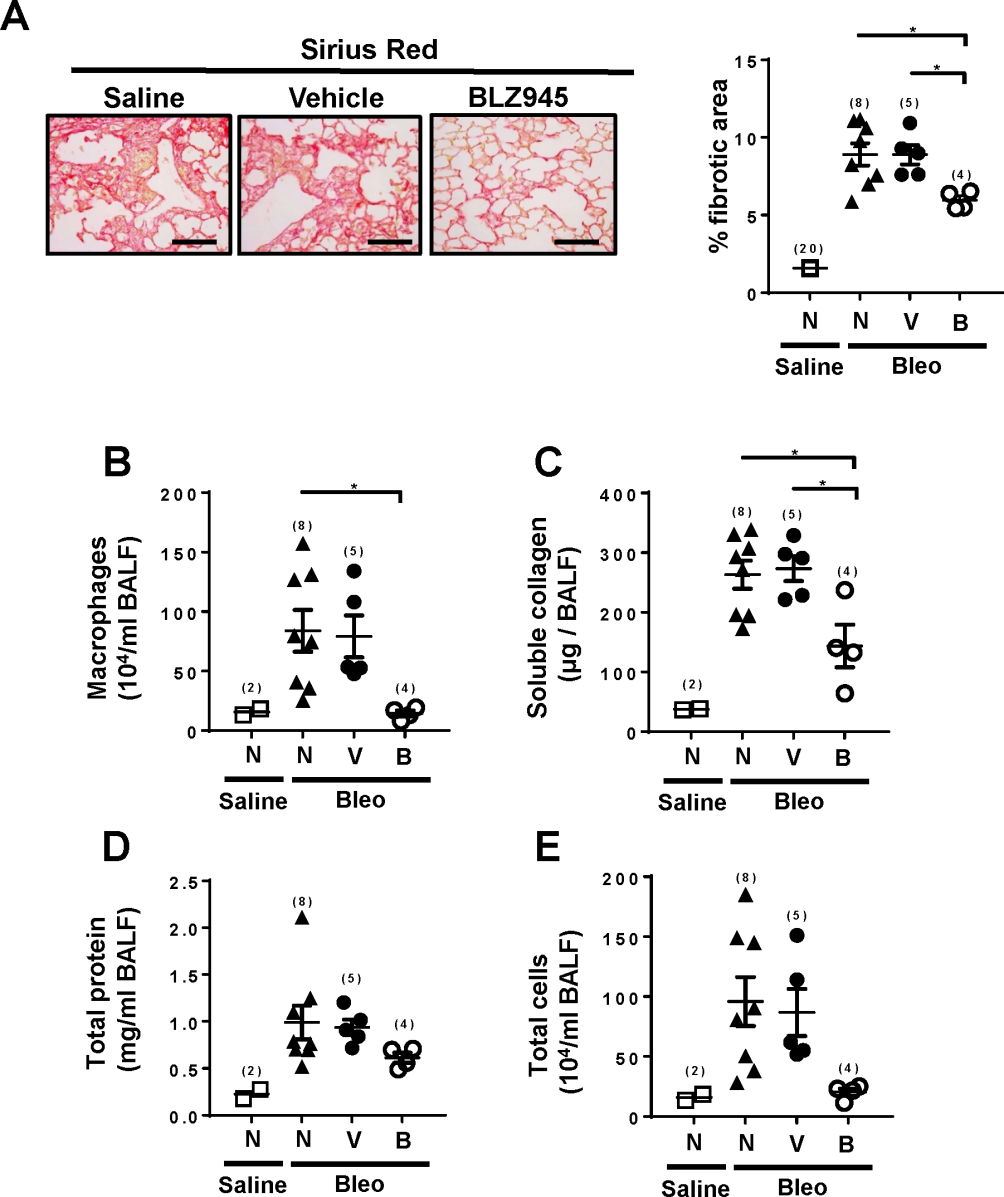
A CSF-1 receptor inhibitor inhibits bleomycin–induced lung fibrosis in mice. The CSF-1 receptor inhibitor BLZ945 (200 mg/kg, open circles), vehicle (20% Captisol) (closed circles) or saline (None group, closed triangles) were administered into wild-type mice given bleomycin once daily by oral gavage. Control wild-type mice (closed square) received i.p. injection of saline. The lungs (A) and BALF (B-E) were collected on day 25 after starting bleomycin administration and analyzed. (A) Representative tissue images of Sirius Red–stained lung sections (Left). Magnification, x400. Scale bar, 100 μm. Quantification of fibrotic areas in Sirius Red–stained lung sections (Right). N, none; V, vehicle; B, BLZ945. (B-E) Macrophage numbers (B), soluble collagen levels (C), total protein concentrations (D), and total cell numbers (E) in BALF. The individual values and the mean ± SEM are shown. N = 4−7 from the single experiments, except the saline-administered wild-type control mice in which N was 20 from multiple experiments in (A) and 2 in (B-E). *P <0.05 (one-way ANOVA followed by the Bonferroni post-test).

Although previous studies [30] showed that macrophage depletion resulted in inhibition of lung fibrosis in intratracheal single bleomycin administration models, an involvement of macrophages in a repeated bleomycin injection model, which generates persistent and more diffuse fibrotic lesions differently from intratracheal single bleomycin administration models [29], is unknown. We tested the effects of the specific inhibitor (BLZ945) of the receptor for colony–stimulating factor-1 (CSF-1), which is a growth factor necessary for differentiation, proliferation and survival of macrophages [23]. BLZ945 inhibited lung fibrosis with depletion of alveolar macrophages in BALF whereas the vehicle did not affect fibrosis (Fig 4A and 4B). BLZ945 also reduced the levels in BALF of soluble collagen, total proteins and total cells (Fig 4C–4E). Thus, depletion of macrophages suppressed bleomycin–induced lung fibrosis. These results, together with the data in BM chimera experiments, suggest that S1PR2 in macrophages may be involved in bleomycin–induced lung fibrosis.

### *S1pr2* deletion suppresses the expression of profibrotic genes in macrophages

**Fig 5:**
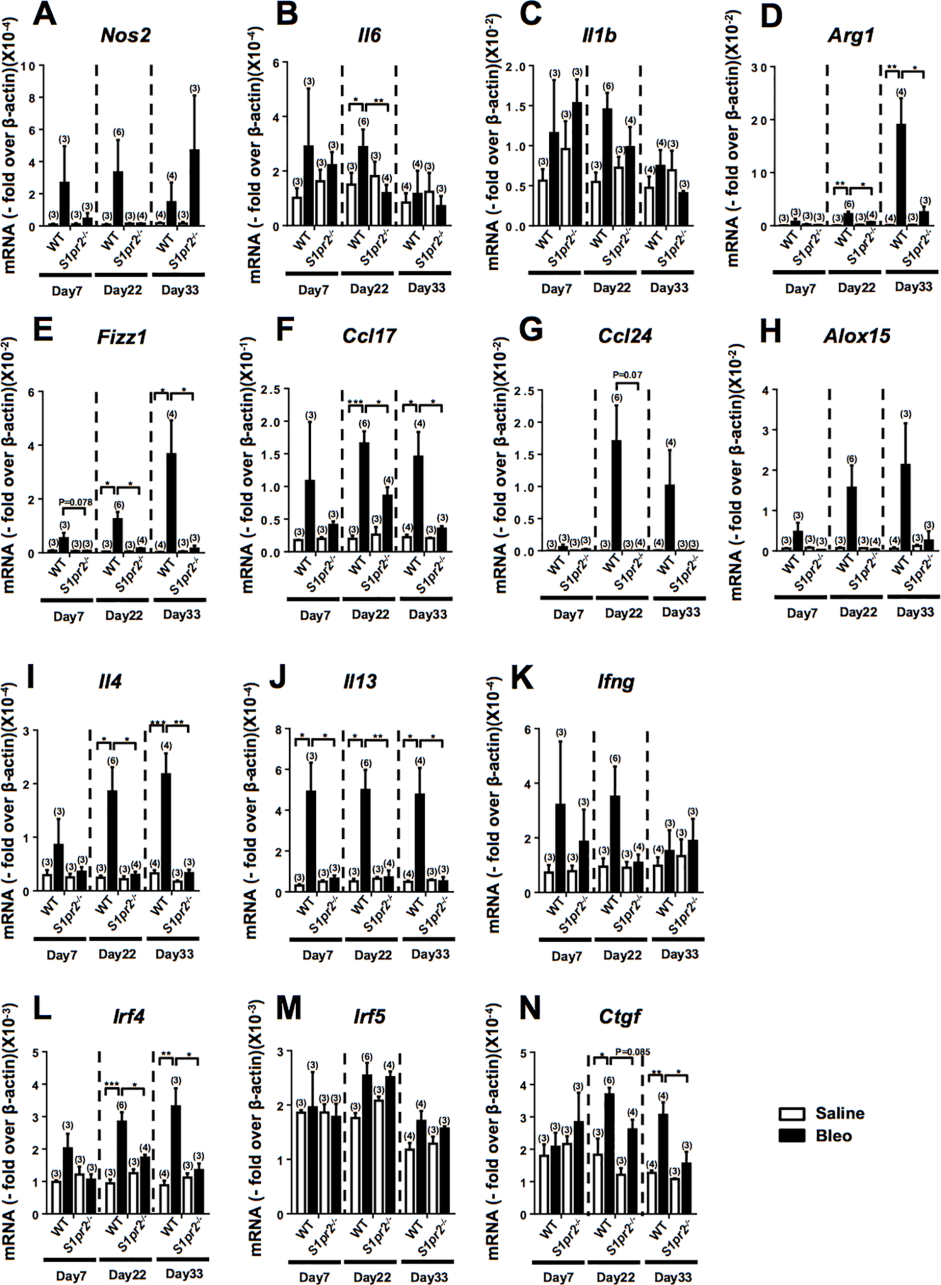
*S1pr2* deletion suppresses the expression of profibrotic genes in BALF cells. The mRNA expression levels of M1 and M2 markers and related genes (A-N) in BALF cells from wild-type (WT) and *S1pr2*^*-/-*^ mice on days 7, 22 and 33 after starting administration of bleomycin or saline were determined by qPCR. The open and solid bars indicate the values of saline–and bleomycin (Bleo)–administered mice, respectively. The individual values and the mean ± SEM are shown. Each sample was prepared from the pooled cells obtained from 3–4 mice. N = 3−6 from three independent experiments. *P <0.05, **P <0.01 and ***P<0.001 (two-way ANOVA followed by the Bonferroni post-test).

We studied the effects of *S1pr2* deletion on mRNA expression in BALF cells using DNA microarray analysis. In BALF cells from bleomycin–administered *S1pr2*^*-/-*^ mice, 398 genes showed downregulation by less than 0.5-fold compared with bleomycin–administered wild-type BALF cells (GEO accession number: GSE85411). In contrast, 122 genes showed upregulation by more than 2.0-fold in *S1pr2*^*-/-*^ BALF cells. The downregulated genes in *S1pr2*^*-/-*^ mice included the genes of profibrotic cytokines, chemokines and the markers characteristic of alternatively activated (M2) macrophages [31, 32] (Table 1). To confirm their differential expression, we determined time–dependent changes in mRNA levels in BALF cells from bleomycin–or saline–administered wild-type and *S1pr2*^*-/-*^ mice, using qPCR. Bleomycin increased the expression of the profibrotic genes including *Il4*, *Il13*, *Irf4* and *Ctgf* in wild-type BALF cells on days 22 and 33 (Fig 5I, 5J, 5L and 5N). Bleomycin did not increase mRNA expression of the powerful profibrotic factor TGFβ1 in either wild-type or *S1pr2*^*-/-*^ cells (data not shown). Bleomycin also increased the expression of the “M2 marker” genes including *Arg1*, *Fizz1*/*Resistin like alpha (Retnla)*/*Resistin–like molecule α (RELMa)*, *Ccl17*, *Ccl24* and *Alox15*. *S1pr2* deletion markedly attenuated or abolished bleomycin–induced increases in the mRNA expression of the profibrotic genes as well as M2 marker genes (Fig 5D–5H). Bleomycin also tended to increase the expression of some M1 marker genes including *Nos2*, *Il6* and *Il1b* in wild-type BALF cells (Fig 5A–5C).

**Table 1:** List of chemokines, cytokines, macrophage-related and extracellular matrix-related genes upregulated by more than 2.0-fold and downregulated by less than 0.5-fold in *S1pr2*^*-/-*^ BALF cells relative to wild-type BALF cells from bleomycin-treated mice by microarray analysis.

	Genes	Accession #	Increase	Decrease
Chemokine and their receptor genes				
	*Cxcl13*	NM_018866	2.61	
	*Ccl24*	NM_019577		0.0033
	*Ccr3*	NM_009914		0.045
	*Cxcl3*	NM_203320		0.31
	*Ccr4*	NM_009916		0.33
	*Ccl4*	NM_013652		0.36
	*Ccl17*	NM_011332		0.44
	*Ccr10*	NM_007721		0.47
	*Ccl8*	NM_021443		0.49
Cytokine and their receptor genes and related genes				
	*Il13*	NM_008355		0.055
	*Il5ra*	NM_008370		0.075
	*Il4*	NM_021283		0.084
	*Il6*	NM_031168		0.3
	*Socs1*	NM_009896		0.3
	*Il1rl1*	NM_001025602		0.31
	*Il1b*	NM_008361		0.35
	*Cish*	NM_009895		0.48
	*Tnfsf14*	NM_019418		0.49
Macrophage-related genes				
	*Alox15*	NM_009660		0.035
	*Retnla*	NM_020509		0.12
	*Csf1*	NM_007778		0.39
	*Ifng*	NM_008337		0.4
	*Csf1*	NM_001113530		0.46
	*Nos2*	NM_010927		0.48
	*Csf1*	NM_001113530		0.48
Extracellular matrix-related genes				
	*Fgf1*	NM_010197	2.61	
	*Mmp25*	NM_001033339		0.28
	*Mmp9*	NM_013599		0.29
	*Fgfr1*	NM_010206		0.46

**Fig 6:**
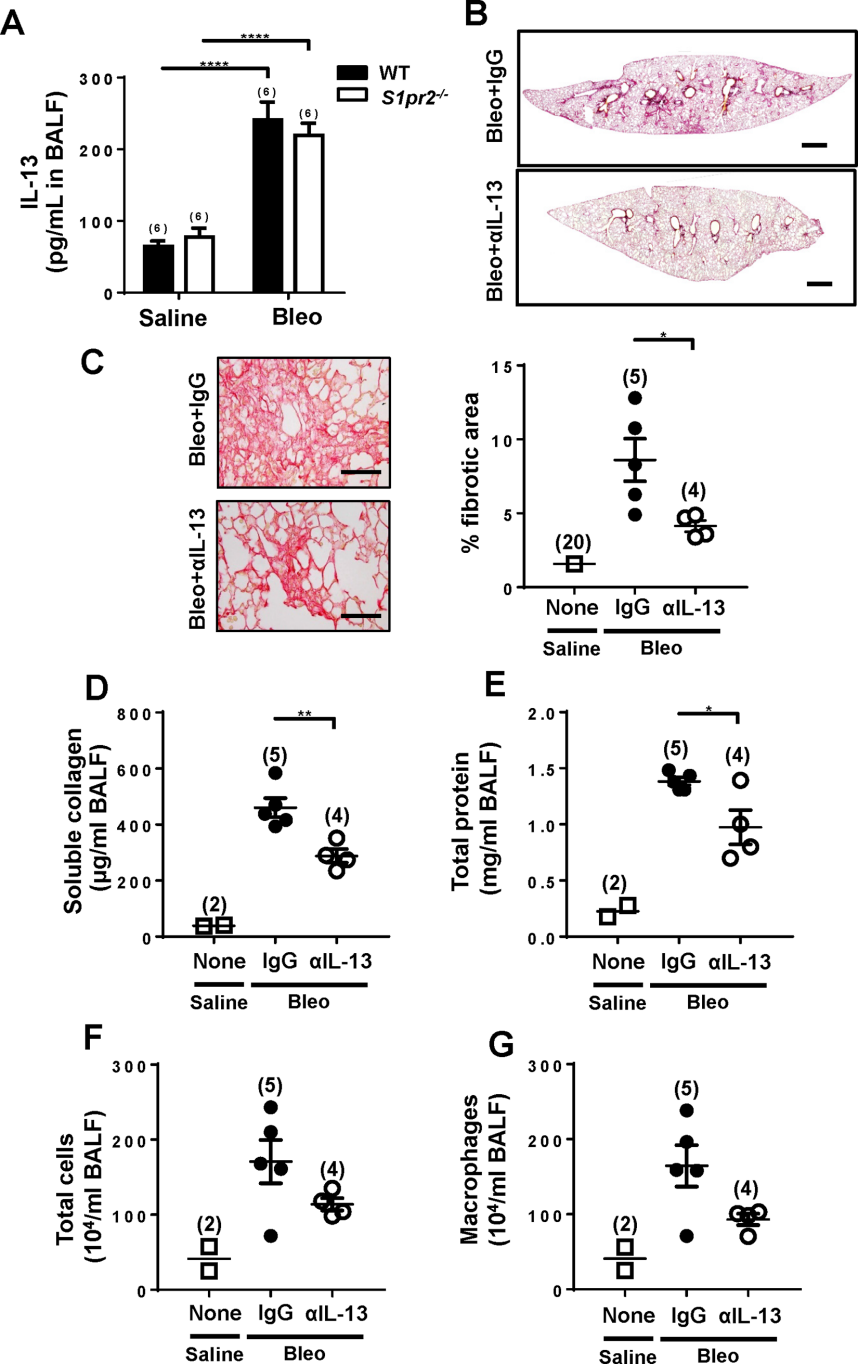
Administration of IL-13–neutralizing antibody ameliorates bleomycin–induced lung fibrosis. (A) IL-13 protein levels in BALF. IL-13 levels in BALF from wild-type (WT) and *S1pr2*^*-/-*^ mice on day 22 after starting saline or bleomycin (Bleo) administration were measured by ELISA. The solid and open bars indicate the values of WT and *S1pr2*^*-/-*^ mice, respectively. In (B)-(G), the mice received intraperitoneal injection of IL-13–neutralizing antibody (αIL-13) or control IgG (IgG) (5 mg/kg, every 11 day), which was begun 1 day before starting bleomycin (Bleo) administration. The lungs and BALF were collected from mice on day 33 and analyzed. (B) Representative images at low magnification (x40) of Sirius Red–stained whole lung sections from wild-type mice given αIL-13 or IgG. Scale bar, 600 μm. (C) Left: Representative images at high magnification (x400) of Sirius Red–stained lung sections. Scale bar, 100 μm. Right: Quantification of fibrotic areas in whole lung sections. (D-G) Soluble collagen levels (D), total protein concentrations (E), total cell numbers (F), and macrophage numbers (G) in BALF. The individual values and the mean ± SEM are shown. In (A), N = 6. In (C-G), N = 4−5 from the single experiment, except the saline-administered wild-type control mice in which N was 20 from multiple experiments in (C) and 2 in (D-G). *P <0.05, **P <0.01, ****P<0.0001 (two-way ANOVA followed by the Bonferroni post-test in (A) and two-tailed Student’s t-test in (C)-(G)).

Among the cytokines and chemokines that were upregulated by bleomycin administration and suppressed by *S1pr2* deletion (Fig 5 and Table 1), IL-13 has recently emerged as a profibrotic mediator in lung fibrosis [33, 34]. Bleomycin administration increased IL-13 concentrations in BALF in wild-type mice (Fig 6A). However, *S1pr2* deletion did not affect IL-13 concentrations in BALF. We tested a possible involvement of IL-13 in a repeated bleomycin injection model of lung fibrosis by studying the effect of anti-IL-13 antibody administration in mice. Administration of anti-IL-13 antibody into wild-type mice substantially inhibited bleomycin–induced lung fibrosis compared with control IgG (Fig 6B and 6C). Consistent with this, administration of anti–IL-13 antibody reduced the concentration of soluble collagen in BALF (Fig 6D) and tended to decrease total protein concentration, total cells and macrophages (Fig 6E–6G). These results indicate that IL-13 plays a crucial role in bleomycin–induced lung fibrosis.

#### *S1pr2* deletion dampens the response of macrophages to IL-13

**Fig 7:**
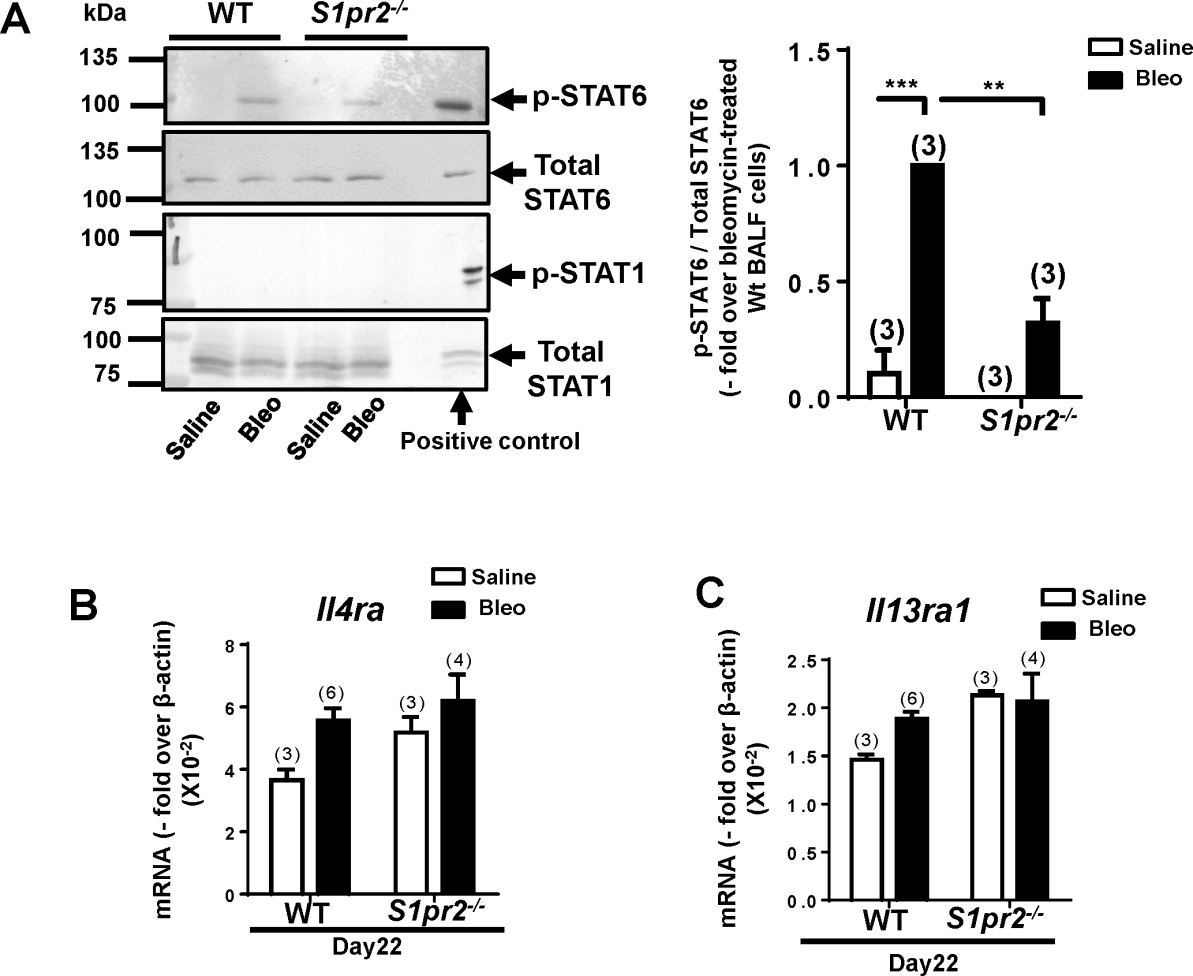
STAT6 activation is suppressed in *S1pr2*^*-/-*^ macrophages. (A) Tyrosine phosphorylation of STAT6 and STAT1 in alveolar macrophages freshly isolated from saline–or bleomycin (Bleo)–administered wild-type (WT) and *S1pr2*^*-/-*^ mice. Left: representative western blots. Right: quantified data. The open and solid bars indicate the values of saline–and Bleo–administered mice, respectively. (B,C) mRNA expression levels of *Il4ra* (B) and *Il13ra1* (C) in BALF cells from WT and *S1pr2*^*-/-*^ mice. The solid and open bars indicate the values of Bleo–and saline–administered mice, respectively. Each sample was prepared from the pooled cells obtained from 8–13 mice. Lysates of IL-13-stimulated BM–derived wild-type macrophages that had been induced to differentiate in the presence of CSF-1 were used as a positive control. The means ± SEM are shown. N = 3−6 from three or four independent experiments. **P <0.01 and ***P<0.001 (two-way ANOVA followed by the Bonferroni post-test).

Macrophages are one of IL-13–target cells [35–37]. IL-13 and IL-4 signal through the receptor molecules IL-4Rα and IL-13Rα1 to specifically phosphorylate and activate STAT6, leading to expression of IL-13/IL-4–regulated genes including *Arg1* and *Fizz1* [38]. We studied phosphorylation of STAT6 in freshly isolated BALF cells. The BALF cells from bleomycin–administered wild-type mice showed an approximately 10-fold increase in the level of STAT6 phosphorylated at Tyr^641^ (p-STAT6) compared with saline–administered control mice (Fig 7A). The level of p-STAT6 was substantially reduced in BALF cells from bleomycin–administered *S1pr2*^*-/-*^ mice compared with wild-type counterpart. In contrast, bleomycin did not increase phosphorylation at Tyr^701^ (p-STAT1) of STAT1, which can be activated by interferon γ, in either wild-type or *S1pr2*^*-/-*^ BALF cells. There was no difference in the mRNA levels of *Il4ra* and *Il13ra1* in BALF cells between wild-type and *S1pr2*^*-/-*^ mice (Fig 7B and 7C), suggesting that the decrease of p-STAT6 in *S1pr2*^*-/-*^ BALF cells was not due to reduced receptor gene expression.

### Pharmacological blockade of S1PR2 attenuates lung fibrosis

**Fig 8:**
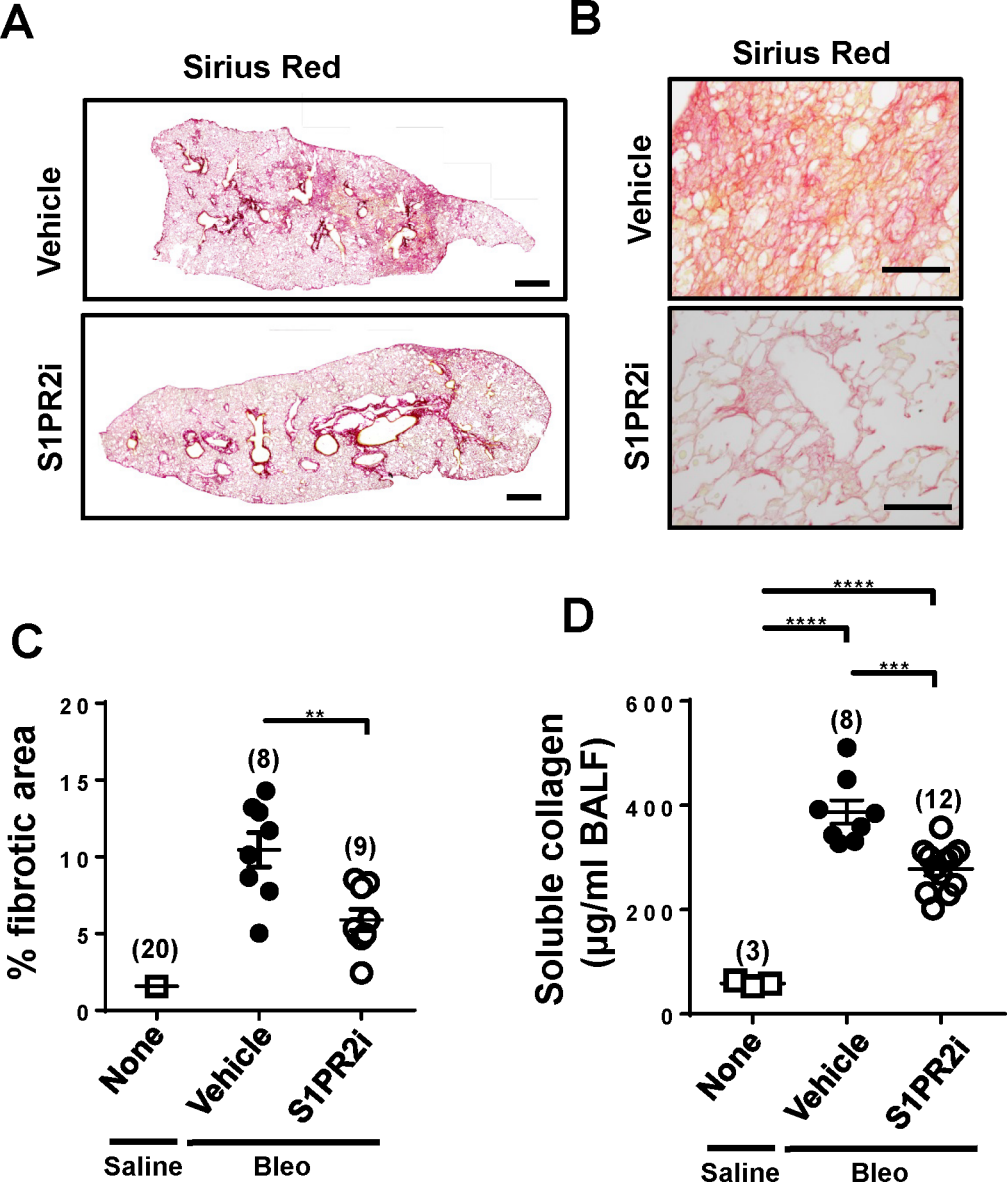
Pharmacological blockade of S1PR2 attenuates lung fibrosis. The S1PR2 antagonist, S1PR2i, was orally administered into wild-type mice, which began 3 days before starting bleomycin administration. The lungs and BALF were collected from mice on day 33 and analyzed. (A) Representative images at low magnification (x40) of Sirius Red–stained whole lung sections from bleomycin–administered wild-type mice given S1PR2i or vehicle. Scale bar: 600 μm. (B) Representative images at high magnification (x400) of Sirius Red–stained lung sections. Scale bar: 100 μm. (C) Quantification of fibrotic areas in whole lung sections. (D) Soluble collagen levels in BALF. The individual values and the mean ± SEM are shown. N = 8−12 from three independent experiments, except the saline-administered wild-type control mice in which N was 20 from multiple experiments in (C) and 3 in (D). **P <0.01, ***P<0.001, ****P<0.0001 (one-way ANOVA followed by the Bonferroni post-test).

We studied the effects of pharmacological blockade of S1PR2 on bleomycin–induced lung fibrosis. The S1PR2–specific antagonist, S1PR2i, was orally administered into bleomycin–treated wild-type mice [18]. S1PR2i suppressed bleomycin–induced lung fibrosis compared with vehicle–treatment (Fig 8A–8C). The level of soluble collagen in BALF from S1PR2i–treated mice was also decreased compared with vehicle–treated wild-type mice (Fig 8D).

## Discussion

The present study first provides evidence for the involvement of S1PR2 in bleomycin–induced lung fibrosis. Second, among several different lung cell types including macrophages, vascular endothelium and fibroblasts that express S1PR2, our studies suggest that S1PR2 in BM–derived cells, most likely in macrophages, contributes to lung fibrosis. Third, mechanistically, S1PR2 is engaged in the production of the profibrotic cytokine IL-13 and the STAT6 phosphorylation response in pulmonary macrophages upon bleomycin administration. Fourth, pharmacological blockade of S1PR2 alleviated bleomycin–induced lung fibrosis, suggesting that S1PR2 is a new promising target for treating lung fibrosis.

Macrophages are engaged in fibrogenesis by secreting profibrogenic factors that recruit and activate fibroblasts and differentiate them into myofibroblasts [30, 32]. TGFβ is one of the best characterized profibrotic cytokines and considered a key player in the pathogenesis of fibrosis. However, we did not find any differences in the protein levels of TGFβ1 in BALF and lung between wild-type and *S1pr2*^*-/-*^ mice. Besides secreting TGFβ, macrophages are an important source of yet other factors that regulate fibrosis, which include PDGF, IGF-1, CTGF and osteopontin [37, 39, 40]. These factors act on fibroblasts to stimulate proliferation, survival and migration, and to promote collagen synthesis. However, DNA microarray data showed no apparent differences in the mRNA expression of these factors between wild-type and *S1pr2*^*-/-*^ BALF cells, except CTGF, which was slightly diminished in *S1pr2*^*-/-*^ BALF cells compared with wild-type BALF cells.

IL-13 and IL-4 have emerged as key mediators of inflammation–and infection–driven fibrosis. Previous studies [34, 41, 42] showed that bleomycin–induced lung fibrosis was diminished in mice deficient in IL-13, IL-4 and their downstream transcription factor STAT6 or treated with a neutralizing anti–IL-13 antibody. In these previous investigations, single bleomycin intratracheal administration protocols were employed. In contrast, our study used repeated bleomycin i.p. administration protocol, which generates substantial differences in progression, persistence and distribution of lung fibrotic lesions from single intratracheal administration models [22, 29]. Our data indicate that IL-13 is a key driver for the repeated injection model of lung fibrosis (Fig 6). IL-13 and IL-4 in part share the receptors and the downstream signaling pathway to drive the differentiation of macrophages toward the phenotype known as alternative activation or M2 activation, which is characterized by upregulated expression of the genes including FIZZ1 and arginase 1, [37, 43]. The increased expression of these genes is mediated largely through activation of STAT6. Among these molecules, the secreted protein FIZZ1 was shown to act on fibroblasts and induce differentiation into myofibroblasts and stimulation of collagen synthesis [44].

Bleomycin–induced stimulation of IL-13/IL-4 expression in BALF cells was markedly reduced in *S1pr2*^*-/-*^ BALF cells. Although T helper 2 (Th2) cells were initially implicated as the major source of IL-13 in the development of fibrosis [37], type II innate lymphoid cells (ILC2s) of the innate immune cell members are more recently emerging as an important source of IL-13 [35]. In addition, recent studies showed that IL-33, an IL-1 family member, activates macrophages to stimulate IL-13 expression [45, 46]. However, in the present study, bleomycin induced the similar extents of increases in IL-13 protein level in BALF (Fig 6A) in wild-type and *S1pr2*^*-/-*^ mice in the face of the markedly reduced IL-13 mRNA level in *S1pr2*^*-/-*^ BALF cells (Fig 5J). These data may suggest that ILC2s and Th2 cells rather than macrophages are a major source of IL-13 in the lung in a repeated bleomycin injection model.

Activation of STAT6, a downstream essential effector of IL-13/IL-4, in BALF cells was 70% reduced in bleomycin–administered *S1pr2*^*-/-*^ mice compared with bleomycin–administered wild-type mice (Fig 7A). Because IL-13 protein levels were similar in BALFs of wild-type and *S1pr2*^*-/-*^ mice, these observations suggest that S1PR2 may be involved in lung fibrosis mainly through potentiating cellular responses to the key profibrotic mediator IL-13 rather than stimulating the production of IL-13 in lung. In cytokine signaling, tyrosine phosphorylation of STAT is catalyzed by Janus kinases and also negatively regulated by tyrosine phosphatases and suppressor of cytokine signaling (SOCS) proteins [47–49]. The regulation of STAT6–specific tyrosine phosphatases, which include SHP-1 and PTP1B, is not well understood and the possibility of tyrosine phosphatase inhibition by S1PR2 remains to be clarified in future studies. In the present study, we observed changes in the expression of some SOCS members in *S1pr2*^*-/-*^ BALF cells (Table 1). In any event, these observations collectively suggest that *S1pr2* deletion in BALF cells impedes STAT6 activation and thereby inhibits the expression of STAT6–targeted profibrotic genes including *Fizz1*, leading to the attenuation of lung fibrosis.

S1PRs seem to exert receptor subtype–specific distinct effects on lung fibrosis. In contrast to S1PR2 blockade, the functional antagonism of S1PR1 by continuous administration of an S1PR1 agonist, which brought about robust S1PR1 internalization and consequent downregulation of cell surface S1PR1 in the vascular endothelium, led to aggravation of lung fibrosis due to impaired vascular barrier integrity [5,11]. In this point, it is notable that the major site of S1PR2 action in lung fibrosis is likely BM–derived cells. Also, the signaling property of S1PR2 is distinct from that of S1PR1: S1PR1 is exclusively coupled to G_i_ signaling pathway whereas S1PR2 is coupled to G_12/13_ pathway [5]. More recently, *S1pr3* deletion was reported to attenuate bleomycin–induced lung fibrosis [12]. However, the S1PR3–expressing cell type that plays a crucial role in lung fibrosis, and the critical intracellular signaling pathway downstream of S1PR3 are unknown.

In summary, our study demonstrates a novel role for S1PR2 in regulating cellular phenotypes of BM–derived cells, particularly macrophage, and resultantly fibrogenesis during the development of bleomycin–induced pulmonary fibrosis. The profibrotic effects of S1PR2 are suggested to be mediated largely through augmented responses of the cells including macrophages to IL-13. This work also suggests that targeting S1PR2 offers a new therapeutic strategy for IPF.

## Supporting information

S1 Table List of primer pairs used in qPCR. (PDF)
