# Supplementary material for: Sphingosine-1-phosphate receptor-2 facilitates pulmonary fibrosis through potentiating IL-13 pathway in macrophages
Source: PLoS One. 2018 May 21;13(5):e0197604. doi: 10.1371/journal.pone.0197604 (PMC5962071; doi:10.1371/journal.pone.0197604)
Supplement: S1 Table — (PDF) [file pone.0197604.s001.pdf]

S1 Table. List of primer pairs used in qPCR

Genes	Primer pair sequences (forward/reverse)
Fibronectin	5’-tctgggaaatggaaaaggggaatgg-3’<br>5’-cactgaagcaggtttcctcggttgt-3’
Collagen1a1	5’-cctggcaaagacggactcaac-3’<br>5’-gctgaagtcataaccgccactg-3’
Nos2	5’-ctgcagcacttggatcaggaacctg-3’<br>5’-gggagtagcctgtgtgcacctggaa-3’
Il6	5’-ccggagaggagacttcacag-3’<br>5’-tccacgatttcccagagaac-3’
Il1b	5’-caggcaggcagtatcactca-3’<br>5’-aggccacaggtattttgtcg-3’
Arg1	5’-aagacagcagaggaggtgaagag-3’<br>5’-tgggaggagaaggcgtttgc-3’
Fizz1	5’-ttgcaactgcctgtgcttac-3’<br>5’-caagaagcagggtaaatggg-3’
Ccl17	5’-agtggagtgttccagggatg-3’<br>5’-ctggtcacaggccgttttat-3’
Ccl24	5’-ccaagaagggccataagatctg-3’<br>5’-gcccctttagaaggctggtt-3’
Alox15	5’-cagggatcggagtacacgtt-3’<br>5’-gattgtgccatccttccagt-3’
Il4	5’-acaggagaagggacgccat-3’<br>5’-gaagccctacagacgagctca-3’
Il13	5’-gcttattgaggagctgagcaaca-3’<br>5’-ggccaggtccacactccata-3’
Ifng	5’-tggctctgcaggattttcat-3’<br>5’-tcaagtggcatagatgtgga-3’
Irf4	5’-tccgacagtggttgatcgac-3’<br>5’-cctcacgattgtagtcctgctt-3’
Irf5	5’-ggtcaacggggaaaagaaact-3’<br>5’-catccaccccttcagtgtact-3’
Ctgf	5’-agcctcaaactccaaacacc-3’<br>5’-caacagggatttgaccac-3’
Il4ra	5’-gagtgagtggagtcctagcatc-3’<br>5’-gctgaagtaacagaacaggc-3’
Il13ra1	5’-gaatttgagcgtctctgtcgaa-3’<br>5’-ggttatgccaaatgcacttgag-3’
Actb	5’-aggtcatcactattggcaacga-3’<br>5’-cacttcatgatggaattgaatgtagtt-3’
